# Shaping Plant Adaptation in a Warmer World: Developmental Plasticity Under Moderately Elevated Temperatures

**DOI:** 10.3390/plants15152410

**Published:** 2026-08-06

**Authors:** Junfeng Zhai, Xin Liu, Xiaobin Sun, Ruize Han, Jiewei Zhang, Yan Liang

**Affiliations:** 1 State Key Laboratory of Wheat Improvement, Shandong Agricultural University, Tai’an 271018, China; sndzjf@163.com (J.Z.); 18363381840@163.com (X.L.); 18538931915@163.com (X.S.); hanruize2023@163.com (R.H.); 2 Beijing Key Laboratory of Crop Molecular Design and Intelligent Breeding, Beijing Academy of Agriculture and Forestry Sciences, Beijing 100097, China; zhangjiewei@baafs.net.cn

**Keywords:** moderately elevated temperatures, developmental plasticity, vegetative development, reproductive development

## Abstract

As global warming raises ambient temperatures, plant growth and productivity are being profoundly affected. Although the impacts of extreme heat stress have received extensive attention, the developmental consequences of moderately elevated temperatures remain less understood. As sessile organisms, plants rely on developmental plasticity to adjust their growth and development in response to warm environments. Understanding how plants sense temperature and convert this signal into developmental outputs is crucial for determining their adaptive strategies to climate change and for applying this knowledge to breed climate-resilient crops. Here, we review current advances in understanding how moderately elevated temperatures regulate developmental plasticity throughout the plant life cycle, including the perception of moderate warmth, the resulting developmental plasticity during vegetative and reproductive growth, and the coordination and trade-offs between these two phases. We also highlight major unanswered questions in this field and propose strategies for manipulating developmental plasticity to breed climate-resilient crops.

## 1. Introduction

Throughout their life cycle, plants are constantly exposed to fluctuating environmental conditions [1]. Among various environmental factors, ambient temperature stands out as one of the most influential signals [2]. Over recent decades, a gradual warming trend has subjected plants to persistently higher ambient temperatures across the globe [3]. These temperature changes can profoundly affect plant growth and development, thereby altering species distribution and reducing crop yield [4]. Relative to the 1850–1900 baseline, the average global surface temperature from 2011 to 2020 rose by 1.09 °C (with a likely range of 0.95 °C to 1.20 °C) [5]. It is estimated that each 1 °C increase in temperature leads to yield losses of approximately 6.0% for wheat (*Triticum aestivum* L.), 3.2% for maize (*Zea mays* L.), and 7.4% for rice (*Oryza sativa* L.) [6,7], posing a substantial threat to global food security.

Global warming alters plant thermal environments mainly through two aspects: the increased frequency of extreme heat events and the gradual elevation of ambient temperatures [3]. Extreme heat refers to temperatures exceeding thresholds, which cause irreversible damage and may ultimately lead to plant death [8]. In contrast, moderately elevated ambient temperatures refer to temperature increases above the optimal growth temperature but below the threshold that causes irreversible heat injury [9]. Due to substantial variation in thermal optima and heat tolerance among plant species, genotypes, and developmental stages, moderately elevated temperatures and extreme heat stress cannot be defined by a single universal threshold. Therefore, representative temperature regimes frequently adopted in experimental studies of moderate warming responses and extreme heat stress across different plant systems are summarized in Table 1.

In this review, we focus on moderately elevated ambient temperatures. As sessile organisms, plants cannot move to favorable environments when exposed to moderately elevated ambient temperatures. Instead, they rely on remarkable developmental plasticity to optimize growth, development, and reproduction under warmer conditions [30]. This plasticity is not a passive response but rather an adaptive strategy shaped by evolution. Elucidating the molecular basis of how plants, especially crops, respond to and tolerate moderately elevated ambient temperatures would help address global food security issues posed by climate warming.

## 2. Thermal Perception of Moderately Elevated Temperature

**Figure 1:**
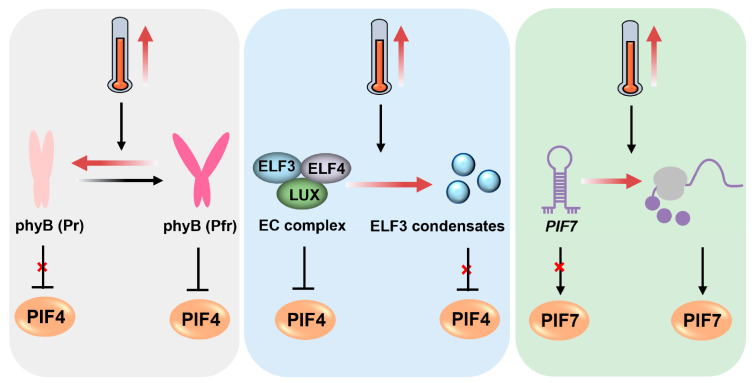
Mechanisms underlying plant perception of moderately elevated temperatures. Plants sense moderately elevated temperatures through multiple thermosensory mechanisms that converge to regulate the abundance of PIF4 and PIF7. Left panel: phyB functions as a thermosensor through temperature-accelerated reversion from the active Pfr form to the inactive Pr form, thereby relieving repression of PIF4 [31,32,33,34,35,36,37]. Middle panel: moderately elevated temperature induces phase separation and condensate formation of ELF3, leading to disruption of the Evening Complex and derepression of *PIF4* expression [38,39,40,41,42]. Right panel: moderately elevated temperature promotes unfolding of the *PIF7* mRNA hairpin structure, enhancing *PIF7* translation [43]. Arrows denote positive regulation, and T-bars denote negative regulation. Abbreviations: phyB, PHYTOCHROME B; PIF4, PHYTOCHROME INTERACTING FACTOR 4; PIF7, PHYTOCHROME INTERACTING FACTOR 7; ELF3, EARLY FLOWERING 3; EC, Evening Complex; ELF4, EARLY FLOWERING 4; LUX, LUX ARRHYTHMO.

Precise perception of temperature fluctuations is a prerequisite for plants to respond to moderately elevated ambient temperatures. To date, multiple thermosensory mechanisms have been identified in plants, which ultimately converge on downstream transcriptional regulators, particularly members of the PHYTOCHROME INTERACTING FACTOR (PIF) family, to initiate thermoresponsive developmental programs (Figure 1) (Table 2).

One such mechanism involves phytochrome B (phyB), which functions as a dual sensor for both light and temperature [31]. PhyB exists in two photoconvertible conformations, Pr (the inactive form) and Pfr (the active form). Red light converts Pr to Pfr, whereas far-red light triggers the reverse conversion from Pfr to Pr [32,33]. The active Pfr state of phyB promotes the inactivation of the PIF family transcription factors, such as PIF4 [34,35]. In *Arabidopsis*, moderate increases in ambient temperature (typically from approximately 22 °C to 27–28 °C) enhance the thermal reversion of the active phyB Pfr form to the inactive Pr form, thereby relieving the repression of PIFs, which ultimately activates downstream thermoresponsive gene expression [36,37].

A distinct thermosensor is EARLY FLOWERING 3 (ELF3). Together with ELF4 and LUX ARRHYTHMO (LUX), ELF3 forms the Evening Complex, which directly represses *PIF4* expression [38,39]. ELF3 possesses a polyglutamine (polyQ) repeat located within a prion-like domain. Under moderately elevated temperatures, this domain undergoes liquid–liquid phase separation, forming reversible condensates that disrupt the interaction between ELF3 and its partners ELF4 and LUX. This impairment of the evening complex assembly relieves the transcriptional repression of *PIF4* [40,41,42].

An additional layer of thermosensing operates at the RNA level through direct regulation of *PIF7*, another key member of the PIF family involved in temperature-responsive growth. In *Arabidopsis*, a thermosensitive RNA hairpin structure located in the 5′ untranslated region (UTR) of *PIF7* undergoes a conformational change under moderately elevated temperatures, which markedly enhances the translation efficiency of *PIF7* mRNA and thereby promotes thermoresponsive growth [43].

Collectively, these thermosensory pathways channel temperature signals toward key transcriptional regulators such as PIFs, which then integrate environmental and endogenous cues to orchestrate developmental plasticity.

## 3. Developmental Plasticity Under Moderately Elevated Temperatures

Once thermal signals are perceived, plants exhibit developmental plasticity throughout their life cycle. This plasticity is defined as the ability of a given genotype to generate different phenotypes in response to environmental cues [76]. It represents a core adaptive strategy that plants employ to cope with moderately elevated temperatures, operating throughout the entire life cycle rather than being confined to any single developmental phase. During the vegetative phase, moderately elevated temperatures trigger pronounced and generally adaptive developmental plasticity, manifesting as alterations in morphological architecture and developmental progression that collectively reduce heat capacity and optimize resource acquisition [9,77]. In addition, moderately elevated temperatures promote flowering, thus initiating the transition from vegetative to reproductive growth [45]. During the reproductive phase, plants still retain some degree of developmental plasticity under moderately elevated temperatures. However, this plasticity is subject to tighter constraints to maintain the stability of key agronomic traits, such as spike morphology, grain quality, and yield [25,74,78]. We review the developmental plasticity responses in two stages that mirror the natural developmental sequence, beginning with the vegetative growth phase, where we highlight adaptive changes in plant architecture and growth dynamics. This is followed by an examination of the reproductive phase, emphasizing the balance and constraint of developmental plasticity that support continuous reproduction and yield. Finally, we discuss the coordination between vegetative and reproductive developmental plasticity.

### 3.1. Developmental Plasticity During Vegetative Growth

**Figure 2:**
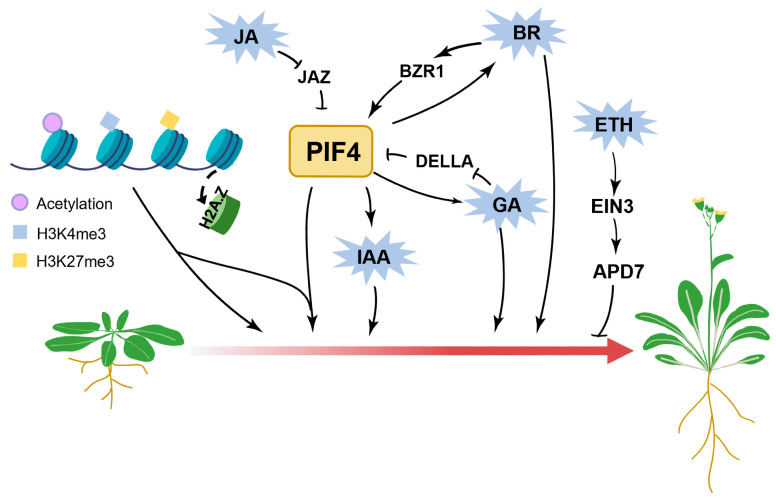
Regulation of vegetative developmental plasticity under moderately elevated temperatures. Under moderately elevated temperatures, plants exhibit adaptive developmental plasticity during vegetative growth, such as hypocotyl and petiole elongation, leaf hyponasty, enhanced primary root elongation, and early flowering. These plastic responses are governed by multiple integrated pathways. PIF4 functions as a central integrator of thermomorphogenic signaling and coordinates downstream growth responses [47,48,49,50,86]. Epigenetic regulation (H2A.Z dynamics, histone methylation, and acetylation) modulates the transcription of thermoresponsive genes, acting in part cooperatively with PIF4 [52,53,54,55,56,57,58,59,61,62,87,88]. In parallel, multiple phytohormone pathways (IAA, BR, GA, JA, and ETH) extensively interact with PIF4-mediated signaling through complex regulatory crosstalk [63,64,65,66,67,68,69,70,71,72,73,89,90,91,92]. Arrows denote positive regulation, and T-bars denote negative regulation. Abbreviations: PIF4, PHYTOCHROME INTERACTING FACTOR 4; IAA, indole-3 acetic acid; BR, brassinosteroid; GA, gibberellin; JA, jasmonic acid; ETH, ethylene; BZR1, BRASSINAZOLE RESISTANT 1; JAZ, JASMONATE-ZIM DOMAIN; EIN3, ETHYLENE INSENSITIVE 3; APD7, ARABIDOPSIS PP2C CLADE D7.

During vegetative growth, plants display adaptive developmental plasticity in response to moderately elevated temperatures, characterized by coordinated changes in plant architecture and growth dynamics (Figure 2). Much of our current understanding of this plasticity comes from studies in the dicot model *Arabidopsis thaliana* (Table 2). In *Arabidopsis*, moderately elevated temperatures induce hypocotyl elongation, petiole extension, and leaf hyponasty, which collectively enlarge the ventilation space and reduce heat accumulation caused by leaf overlap [79,80]. Concurrently, leaves exhibit further plastic adjustments by producing fewer leaves, reducing individual leaf size, and decreasing leaf thickness, all of which lower overall heat capacity [81]. Stomata, as key structures linking photosynthesis and water metabolism, show reduced density under moderately elevated temperature, thereby preventing excessive water loss [46]. Below ground, the root system displays equally marked plasticity: primary root elongation is promoted and lateral root development is accelerated, facilitating avoidance of topsoil drought and enhancing the capture of water and nutrients from deeper soil layers [82,83]. These plastic responses are broadly conserved across species. For instance, moderately elevated temperatures promote hypocotyl elongation in soybean (*Glycine max* (L.) Merrill), tomato (*Solanum lycopersicum* L.), and cabbage (*Brassica oleracea* var. *capitata* L.), and leaf hyponasty is prominently observed in tomato [60,84]. In monocots, moderately elevated temperatures typically affect growth rates, including leaf elongation and the length of leaf internodes and leaf sheaths [85].

#### 3.1.1. PIFs Coordinate Temperature-Induced Developmental Plasticity

PIFs act as key downstream integrators that link temperature perception to developmental plasticity. Among the PIF family members, PIF4 has been extensively characterized as a central integrator linking temperature perception to developmental plasticity during vegetative growth [80,93,94,95] (Figure 2). Moderately elevated temperatures enhance both the transcription and stability of PIF4, leading to its accumulation [86]. Subsequently, PIF4 serves as a master switch to initiate thermomorphogenic responses by promoting the expression of target genes [47,48,49]. These target genes are involved in hormone biosynthesis, developmental switches, and cell structure [93].

PIF4 promotes hypocotyl elongation through multiple coordinated mechanisms. Auxin plays a central role in this process. PIF4 directly binds to the promoters of *YUCCA8* (*YUC8*), *TRYPTOPHAN AMINOTRANSFERASE OF ARABIDOPSIS 1* (*TAA1*), and *CYTOCHROME P450 CYP79B2*, thereby activating distinct auxin biosynthetic pathways [47,48]. Elevated auxin levels induce the expression of *SMALL AUXIN UP RNA* (*SAUR*) genes, which in turn drives hypocotyl elongation [48]. In parallel with auxin biosynthesis, PIF4 also modulates auxin sensitivity through microRNA156 (miR156) and its target SQUAMOSA PROMOTER-BINDING-PROTEIN-LIKE 9 (SPL9) module, which serves as a critical downstream regulatory node in PIF4-mediated thermomorphogenesis. Disruption of miR156 function abolishes the hypocotyl elongation response to moderately elevated temperatures in *Arabidopsis* [86]. Additionally, PIF4 regulates microtubule reorganization to promote cell elongation. Under moderately elevated temperatures, SPIRAL1 (SPR1) expression and protein stability are enhanced in a PIF4-dependent pathway. Accumulated SPR1 drives the reorganization of cortical microtubules from an oblique/longitudinal to a transverse orientation, ultimately promoting hypocotyl elongation [49].

Beyond promoting hypocotyl elongation, PIF4 also coordinates other aspects of developmental plasticity. In stomatal development, PIF4 directly represses the transcription of *SPEECHLESS* (*SPCH*), a master regulator that initiates the stomatal lineage, leading to a significantly lower stomatal index in *Arabidopsis* under moderately elevated temperatures. Conversely, SPCH exerts negative feedback by binding to the *PIF4* promoter and repressing its expression, thereby preventing a complete arrest of stomatal development [46]. PIF4 also governs leaf hyponasty. Specifically, it directly binds to the promoter of *PINOID* (*PID*), which encodes a protein kinase that phosphorylates the auxin efflux transporter PIN-FORMED3 (PIN3). PID promotes the polarization of PIN3 to the outer membranes of abaxial petiole cells. This polarized localization directs auxin flow toward the abaxial side of the petiole, establishing an auxin gradient that drives asymmetric cell expansion and upward leaf bending [50]. In the regulation of flowering, PIF4 directly binds to and activates the promoter of *FLOWERING LOCUS T* (*FT*), a key floral pathway integrator, thereby accelerating flowering. This PIF4-mediated regulation of *FT* is temperature-dependent. Moderately elevated temperature reduces H2A.Z nucleosome occupancy in the *FT* promoter region, which increases chromatin accessibility and facilitates PIF4 binding [45].

In addition to PIF4, PIF7 represents another important member of the PIF family involved in temperature-induced developmental plasticity. Under moderately elevated temperatures alone, PIF4 acts as a major regulator of hypocotyl elongation, whereas PIF7 becomes particularly important when moderately elevated temperature coincides with shade signals characterized by reduced red/far-red (R/FR) ratios [51]. Under combined moderately elevated temperature and shade conditions, PIF7-dependent upregulation of auxin target genes leads to substantial auxin accumulation and exaggerated hypocotyl elongation. Importantly, this synergistic response is largely independent of PIF4 activity, suggesting that PIF7 represents a distinct regulatory module specialized for integrating temperature and shade signals rather than simply acting as a redundant component of the PIF4-mediated thermomorphogenic pathway [51].

Collectively, PIF transcription factors function as central downstream integrators that translate temperature signals into developmental plasticity. While PIF4 serves as a primary regulator of classical thermomorphogenic responses, PIF7 provides an additional layer of environmental adaptation by mediating growth adjustments under combined temperature and light conditions.

#### 3.1.2. Epigenetic Regulation of Temperature-Induced Developmental Plasticity

Epigenetic regulation serves as an additional control layer that precisely adjusts developmental plasticity in response to temperature. Rather than acting independently, epigenetic mechanisms function in close coordination with central transcriptional regulators, such as PIF4, to shape thermoresponsive growth (Figure 2).

The histone variant H2A.Z plays a central role in temperature-responsive chromatin remodeling. At low ambient temperatures, H2A.Z is enriched at the transcription start sites (TSSs) of genes to repress transcription, whereas it dissociates from chromatin under moderately elevated temperatures to enable gene expression [52]. The deposition and removal of H2A.Z are tightly controlled by a network of regulators. ACTIN-RELATED PROTEIN 6 (ARP6) promotes the incorporation of H2A.Z into nucleosomes [87]. Moderately elevated temperature triggers the upregulation and activation of MITOGEN-ACTIVATED PROTEIN KINASE 4 (MPK4), which subsequently phosphorylates PIF4. Phosphorylated PIF4 inhibits the expression of *ARP6*, thereby reducing H2A.Z deposition at thermoresponsive loci [53]. Conversely, the INO80 chromatin remodeling complex (INO80-C) interacts with PIF4 and mediates H2A.Z eviction from PIF4 target sites, facilitating the expression of plasticity-related genes [54]. The mediator component MEDIATOR 25 (MED25) directly promotes H2A.Z eviction at the *YUC8* locus by recruiting HISTONE DEACETYLASE 9 (HDA9), thereby enhancing PIF4 binding [56,57].

Beyond regulating chromatin accessibility through H2A.Z dynamics, moderately elevated temperatures also modulate plant developmental plasticity through histone modifications that reinforce thermoresponsive transcriptional programs. The Jumonji C (JmjC) domain proteins JUMONJI 14 (JMJ14) and JMJ15, SEUSS (SEU), and INO80 regulate H3K4me3 levels at thermoresponsive loci, thereby controlling gene activation [54,59,61]. The mediator MED17 promotes H3K4me3 enrichment and RNA Polymerase II recruitment at the promoters of *PIF4*, *YUC8*, *IAA19*, and *IAA29* to activate these genes [58]. In parallel, moderately elevated temperatures induce genome-wide accumulation of the repressive mark H3K27me3 in a VERNALIZATION INSENSITIVE 3-LIKE 1 (VIL1)-dependent manner, contributing to the downregulation of genes suppressed under moderately elevated temperatures [88]. A dynamic interplay between activating and repressive marks further refines this response. Ethylene signaling, LIKE HETEROCHROMATIN PROTEIN 1 (LHP1), and INO80 synergistically regulate the “bivalent chromatin state switching” between H3K4me3 and H3K27me3 at target genes, enabling rapid transcriptional adjustments to moderately elevated temperatures [55]. Additionally, in *Arabidopsis*, the histone methylation reader MORF-RELATED GENE 2 (MRG2) directly interacts with PIF4 and binds to thermoresponsive genes such as *YUC8* and *SAUR19* in a PIF4-dependent manner, facilitating the acetylation of H4K5ac and H3K9ac at these loci and thereby activating their expression [62]. In tomato, HISTONE DEACETYLASE 4 (SlHDA4) plays a crucial role in regulating lateral bud outgrowth under moderately elevated temperatures. SlHDA4 forms a complex with TEOSINTE BRANCHED1/CYCLOIDEA/PROLIFERATING CELL FACTOR (TCP) transcription factor SlTCP15 to repress the expression of the photoreceptor gene *PHYTOCHROME B* (*SlPHYB1*) and the auxin signaling repressor *SlIAA12* by removing H3K9ac at their loci, thereby enhancing auxin biosynthesis and signaling and inhibiting lateral bud growth [19]. Collectively, these epigenetic mechanisms act in concert with PIF4-centered transcriptional networks to modulate developmental plasticity under moderately elevated temperatures.

#### 3.1.3. Hormonal Regulation of Temperature-Induced Developmental Plasticity

Phytohormones act as a central execution layer that integrates temperature signals and orchestrates developmental plasticity. Multiple hormone pathways, including auxin, brassinosteroids, gibberellins, jasmonates, and ethylene, form a complex network that fine-tunes growth responses under moderately elevated temperatures [96] (Figure 2).

Among these, auxin (Indole-3-acetic acid, IAA) plays a dominant role in mediating thermoresponsive growth [60,64]. When temperatures rise, the concentration of IAA increases in plants, primarily driven by the significant induction of auxin biosynthesis genes, such as *YUC8* [47]. Notably, this temperature-induced increase in auxin signaling requires the malectin-like receptor kinase FERONIA (FER) [63]. Inhibiting auxin biosynthesis with kynurenine or yucasin significantly attenuates root plasticity under moderately elevated temperatures [64]. The polar transport of auxin is crucial for achieving localized regulation of auxin concentration. Disruption of auxin transport from the cotyledons to the hypocotyl using the polar transport inhibitor 1-N-naphthylphthalamic acid (NPA) inhibits the hypocotyl elongation response to moderately elevated temperatures [60]. Membrane-localized PIN transporters play a key role in this process. Mutants for *PIN* genes, as well as for the D6 PROTEIN KINASE (D6PK) kinase—which regulates PIN phosphorylation—all exhibit attenuated hypocotyl elongation under moderately elevated temperatures [60]. In roots, moderately elevated temperatures promote the targeting of the PIN2 protein to the plasma membrane via a SORTING NEXIN1 (SNX1)-dependent endosomal trafficking pathway, modulating auxin transport and maintaining optimal hormone levels [65]. Furthermore, PIN1 and PIN4 also contribute to root plasticity. The *pin4* mutant displays excessive primary root elongation, whereas the *pin1* mutant shows inhibited elongation under moderately elevated temperatures [64]. Additionally, PIN-LIKES 6 (PILS6), a putative auxin carrier localized to the endoplasmic reticulum (ER), gates nuclear auxin accumulation and perception. Moderately elevated temperatures decrease PILS6 protein abundance, thereby enhancing nuclear auxin signaling and promoting root growth [66].

Brassinosteroids (BRs) play a critical role in regulating vegetative developmental plasticity under moderately elevated temperatures. When ambient temperatures rise, BR biosynthesis is activated [89]. In *Arabidopsis*, treatment with the BR biosynthesis inhibitor propiconazole (PPZ) abolishes hypocotyl elongation induced by moderately elevated temperatures [67]. BRASSINAZOLE RESISTANT 1 (BZR1), the central transcriptional regulator of BR signaling, forms a complex with PIF4 and the auxin response factor AUXIN RESPONSE FACTOR 6 (ARF6) to promote hypocotyl elongation under moderately elevated temperatures [67,68]. BZR1 also directly activates the *PIF4* promoter, further amplifying the plastic growth response [69]. The stability of BZR1 is modulated by the HECT-domain E3 ubiquitin ligase UBIQUITIN PROTEIN LIGASE 3 (UPL3), which interacts with BZR1 to promote its degradation. BZR1 accumulates in *upl3* mutants, leading to accelerated hypocotyl elongation under moderately elevated temperatures [90]. This regulatory function of BR is conserved in tomato, where SlPIF4 promotes hypocotyl elongation by activating *DWARF4* (*SIDWF4*), a key BR biosynthesis gene [21].

Gibberellin (GA), jasmonic acid (JA), and ethylene also contribute to the regulation of vegetative developmental plasticity. When ambient temperatures rise, the expression of the gibberellin (GA) biosynthesis genes *GIBBERELLIN 20 OXIDASE* (*GA20OX1*) and *GA3OX1* is upregulated in *Arabidopsis* hypocotyls, leading to increased GA levels [91]. DELLA proteins, which are core repressors of GA signaling, interact with PIF4, ARF6, and BZR1 to inhibit their DNA-binding activity [34,68,70,71]. GA triggers the degradation of DELLA proteins, thereby relieving their repression on these transcription factors and promoting plastic growth [92]. In the JA pathway, moderately elevated temperatures induce the accumulation of the JA receptor CORONATINE INSENSITIVE1 (COI1). JA binding to COI1 promotes the degradation of JASMONATE-ZIM DOMAIN (JAZ) repressor proteins, which activates MYC2. MYC2 then regulates MEDIATOR SUBUNIT 17 (MED17) to mediate H3K4me3 modification and transcriptional activation of plasticity-related genes such as *PIF4* [58]. Additionally, JAZ3 interacts with PIF4, inhibiting its transcriptional activation function and reducing its ability to bind downstream gene promoters [73]. Ethylene signaling modulates vegetative plasticity through its central regulator ETHYLENE INSENSITIVE3 (EIN3). Under moderately elevated temperatures, EIN3 directly induces the expression of the protein phosphatase *ARABIDOPSIS PP2C CLADE D7* (*APD7*) to inactivate plasma membrane H^+^-ATPases, thereby attenuating auxin-induced cell expansion [72].

### 3.2. Developmental Plasticity During Reproductive Growth

**Figure 3:**
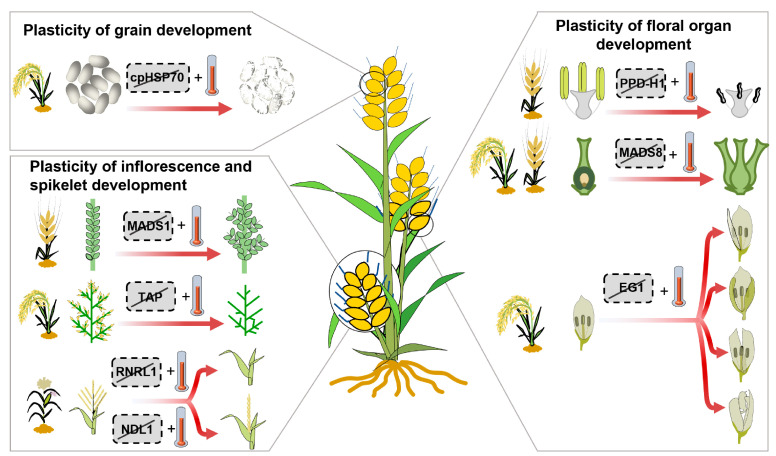
Developmental plasticity during reproductive growth under moderately elevated temperatures. Under moderately elevated temperatures, plants exhibit developmental plasticity during reproductive growth at multiple developmental levels, including inflorescence and spikelet development, floral organ development, and grain development. The boxes with slash marks indicate functional disruption or impairment of the corresponding regulators. Among these, cpHSP70/OsHsp40-1 [74,75], PPD-H1 [26], MADS8 [25], TAP [17], MADS1 [24], and EG1 [15] represent loss-of-function mutants generated by gene knockout or other disruptive mutations, whereas RNRL1 [11] and NDL1 [12] represent point mutants affecting regulator function. Mutation of these regulators leads to enhanced developmental plasticity, resulting in pronounced alterations in inflorescence and spikelet architecture, floral organ identity and determinacy, and grain quality under moderately elevated temperatures. Abbreviations: cpHSP70, CHLOROPLAST HEAT SHOCK PROTEIN 70; PPD-H1, PHOTOPERIOD-H1; MADS1, MCM1-AGAMOUS-DEFICIENS-SRF-box transcription factor 1; TAP, THERMOSENSITIVE BARREN PANICLE; RNRL1, RIBONUCLEOTIDE REDUCTASE LARGE SUBUNIT 1; NDL1, NEEDLE1; EG1, EXTRA GLUME 1.

Moderately elevated temperatures promote flowering, thereby initiating reproductive growth [45]. While vegetative developmental plasticity has been extensively characterized in the model plant *Arabidopsis*, our understanding of reproductive plasticity derives primarily from crop species (Table 2), where reproductive traits are directly linked to agronomic performance. Unlike during vegetative growth, developmental plasticity in reproductive stages is tightly constrained to preserve the stability of reproductive structures and yield. Nevertheless, disruption of key regulatory components releases this latent plasticity, allowing moderately elevated temperatures to drive pronounced phenotypic changes (Figure 3).

#### 3.2.1. Inflorescence and Spikelet Meristem Development

At the early stage of reproductive development, the inflorescence meristem and its derivative spikelet meristems establish the overall architecture of the reproductive structure. During this process, developmental plasticity must be tightly constrained to maintain meristem identity, determinacy, and proliferative activity, thereby preventing temperature-induced reprogramming or developmental arrest. In barley (*Hordeum vulgare* L.), the SEPALLATA-class MCM1-AGAMOUS-DEFICIENS-SRF (MADS)-box protein HvMADS1 constrains spikelet meristem plasticity by maintaining cytokinin homeostasis through activation of the cytokinin oxidase gene *HvCKX3*. Loss of HvMADS1 function removes this constraint, allowing moderately elevated temperatures to reprogram central spikelet meristems into inflorescence meristems and to alter rachilla meristem identity, resulting in ectopic spikelet and spike formation [24]. Similarly, in rice, the transposase-derived FAR1-RELATED SEQUENCE (FRS) protein THERMOSENSITIVE BARREN PANICLE (TAP) constrains the developmental plasticity of inflorescence and spikelet meristems through two complementary mechanisms. On the one hand, TAP directly binds to FRB1 motifs in the promoters of key panicle and spikelet development genes *OsYABBY3* (*OsYAB3*), *OsYAB4*, and *OsYAB5* to activate their expression. On the other hand, TAP forms protein complexes with OsYAB4/5 to regulate meristem development and the expression of floral organ identity genes. Loss of TAP function allows moderately elevated temperatures to induce compromised secondary branch and spikelet initiation [17]. Beyond meristem identity and fate determination, maintenance of metabolic and physiological homeostasis in meristems also constitutes an important constraint on reproductive developmental plasticity. In maize, the mitochondria-localized ATP-dependent metalloprotease NEEDLE1 (NDL1) constrains thermally induced reproductive developmental plasticity by maintaining mitochondrial redox balance and auxin homeostasis within inflorescence meristems. When NDL1 function is compromised, moderately elevated temperatures disrupt axillary meristem initiation and reproductive organogenesis, resulting in severe defects in tassel branching and spikelet formation [12]. Likewise, the ribonucleotide reductase large subunit gene RIBONUCLEOTIDE REDUCTASE LARGE SUBUNIT 1 (ZmRNRL1) is essential for maintaining meristem activity. Loss of ZmRNRL1 disrupts dNTP pools and impairs DNA synthesis, leading to a failure to develop tassels specifically under moderately elevated temperatures [11]. Together, these findings indicate that plasticity during early reproductive development is constrained at multiple levels. When these constraints are compromised, moderately elevated temperatures can trigger either developmental reprogramming or developmental arrest, thereby revealing latent reproductive developmental plasticity.

#### 3.2.2. Floral Meristem and Organ Identity

Following spikelet initiation, the subsequent differentiation of the floral meristem into specific floral organs represents another critical stage at which plasticity is strictly regulated. The conserved MADS-box transcription factor MADS8 in barley and rice restricts floral meristem plasticity through the activation of D-class floral homeotic genes. In the absence of MADS8, moderately elevated temperatures induce a loss of meristem determinacy, leading to the formation of supernumerary carpels [25]. This constraint is further reinforced at the metabolic level by the mitochondrial lipase EXTRA GLUME1 (EG1) in rice. EG1 regulates floral meristem plasticity in a temperature-dependent manner, with its transcription, protein stability, and enzymatic activity all induced by moderately elevated temperatures. Loss of EG1 function enhances the developmental plasticity of floral meristems, which consequently triggers the formation of extra glume-like structures and disrupts floral organ identity under moderately elevated temperatures [15]. Similarly, PHOTOPERIOD 1 (PPD-H1) in barley serves to restrict the developmental plasticity of stamens, ensuring their stable maturation under thermal fluctuations. PPD-H1 regulates the plasticity of stamen and anther development by coordinating and balancing multiple hormonal pathways. Disruption of this balance leads to increased developmental plasticity, manifesting as the formation of stunted, pale anthers under moderately elevated temperatures [26].

#### 3.2.3. Grain Development

Developmental plasticity during reproductive growth is also reflected in grain development. At this stage, the plasticity of grain quality is strictly regulated. The plastid-localized 70 kDa heat shock protein (cpHsp70-2/OsHsp70-2) and its co-chaperone OsHsp40-1 function together as a module that maintains grain quality stability under moderately elevated temperatures. When this chaperone module is intact, grain chalkiness remains relatively stable across different ambient temperatures. However, mutants defective in either component exhibit heightened developmental plasticity in response to moderately elevated temperatures, manifesting as a dramatic increase in chalkiness that is absent at lower temperatures [74,75]. Another study in rice under a moderately elevated night temperature revealed that HSPs, calcium signaling proteins, and nucleic acid/protein modification and repair proteins collectively regulate grain developmental plasticity. These proteins were upregulated to varying degrees during the early grain-filling stage, facilitating the efficient translocation of nonstructural carbohydrate and nitrogen from source to sink, accelerating the grain-filling rates, and maintaining grain weight and quality. Conversely, when such coordinated proteomic reprogramming is lacking, developmental plasticity during rice grain development is enhanced, which results in reduced grain-filling rates and increased chalkiness under moderately elevated temperatures [97].

### 3.3. Coordination Between Vegetative and Reproductive Developmental Plasticity

**Figure 4:**
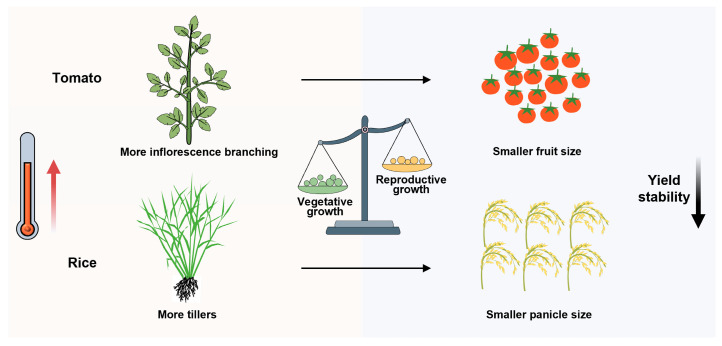
Coordination and trade-offs between vegetative and reproductive developmental plasticity under moderately elevated temperatures. Excessive developmental plasticity during vegetative growth can compromise the potential for reproductive growth and yield stability. For example, under moderately elevated temperatures, tomato (*Solanum lycopersicum* L.) exhibits more inflorescence branches, which lead to reduced fruit size. In rice (*Oryza sativa* L.), moderately elevated temperature increases tiller number, resulting in smaller panicle size.

As discussed above, phenotypic plasticity during vegetative growth enables plants to optimize resource capture and reduce plant body temperature under moderately elevated temperatures. However, excessive morphological adjustment may trigger resource competition and impair the developmental potential of reproductive organs, thereby compromising the stability of reproductive development (Figure 4). In tomato, inflorescence architecture and fruit size represent core agronomic traits governed by a source–sink trade-off. Excessive inflorescence branches lead to resource dilution and smaller fruits, whereas insufficient branches result in an inadequate number of fruits [98]. This plastic response is controlled by the MULTIPLE INFLORESCENCE BRANCH 2 (MIB2)-CONSTANS-Like1 (SlCOL1) transcriptional module. MIB2 is a bHLH transcription factor whose expression is induced in inflorescence meristems by moderately elevated temperatures. MIB2 directly binds to the promoter of *SlCOL1* and activates its transcription. Loss of MIB2 function (as in the *mib2* allele of the cultivated variety Moneymaker) largely suppresses the temperature-induced increase in inflorescence branches and stabilizes fruit size [98]. In cereal crops such as rice and wheat, moderately elevated temperatures often alter tiller number—a key plastic trait determining plant architecture and yield potential [99]. However, a trade-off exists between tiller number and panicle size in rice: when tiller number becomes excessive, panicle size decreases significantly [100,101]. Evidence indicates that moderately elevated temperatures significantly boost tillering, leading to a substantial increase in tiller number, but concurrently reduce grain number per panicle and lower harvest index [102]. Thus, dynamic coordination between the two phases of plasticity is essential for achieving optimal fitness under moderately elevated temperatures.

## 4. Future Perspectives

Against the backdrop of global climate warming, it is crucial to understand how plants rely on developmental plasticity to cope with moderately elevated temperatures. Despite considerable advances, many key questions regarding such plasticity at moderately high temperatures remain unanswered. To begin with, current knowledge of thermosensory mechanisms is still incomplete. It remains largely unknown whether plants possess additional thermosensors, and whether the temperature-sensing pathways identified in *Arabidopsis* are functionally conserved in major crops. Evidence indicates that the rapid thermal reversion property of phyB is retained in both maize and potato (*Solanum tuberosum* L.), pointing to a potentially conserved mechanism across flowering plants [44]. In contrast, while ELF3 homologs are widely present across plant species, their protein structures diverge considerably, especially in the prion-like and polyQ-rich regions implicated in temperature sensing [40]. These findings raise the possibility that different plant species have evolved partially divergent strategies for sensing temperature—an issue that deserves particular attention in crop species. Furthermore, it is still unclear how temperature signals are converted into distinct developmental outcomes depending on the tissues, organs, or developmental stages. Another important question is whether developmental plasticity during vegetative and reproductive growth is governed by a common regulatory framework or by largely independent temperature-responsive networks. Moreover, considering the major differences in body plan between monocots and dicots, it remains largely unexplored whether these two groups have adopted distinct strategies of developmental plasticity in response to moderately elevated temperatures. Addressing these questions will be essential for building a cross-species framework that connects temperature perception, signal transduction, and developmental plasticity.

Another important but largely overlooked question is how moderately elevated temperatures interact with other environmental factors to shape developmental plasticity. In natural and agricultural ecosystems, warming rarely occurs in isolation but is often accompanied by concurrent changes in multiple environmental factors, including water availability, atmospheric CO_2_ concentration, light conditions, nutrient availability, and other abiotic cues. Increasing evidence suggests that these factors can profoundly modify thermoresponsive developmental programs. For example, drought suppresses thermomorphogenesis by promoting salicylic acid accumulation, which inhibits high-temperature-induced ELF3 phase separation and consequently prevents PIF4 activation [103]. Likewise, root elongation in response to high temperature is abolished under low-nitrogen or low-phosphorus conditions, revealing a strong dependence of root thermomorphogenesis on nutrient status [104]. Light-related signals also exert substantial effects on thermoresponsive growth: blue light and UV-B inhibit warm-temperature-induced hypocotyl elongation through CRYPTOCHROME 1 (CRY1) and the UV RESISTANCE LOCUS8 (UVR8)–CONSTITUTIVE PHOTOMORPHOGENIC1 (COP1)–phyB/LONG HYPOCOTYL IN FAR RED 1 (HFR1) module, whereas canopy shade (low R:FR) promotes elongation through PIF7-dependent signaling [51,105,106]. Despite these advances, the molecular mechanisms by which temperature signals are integrated with other environmental cues remain largely unclear. Future studies should move beyond temperature responses in isolation and focus on how multiple environmental signals converge to shape developmental decisions under realistic field conditions.

Addressing these challenges will demand the integration of emerging experimental and computational tools. Advances in artificial intelligence (AI)-driven protein structure prediction and molecular dynamics simulations are expected to accelerate the identification of candidate thermosensors and to uncover the structural underpinnings of temperature sensing [107]. Single-cell and spatial multi-omics approaches will offer opportunities to decipher how temperature-responsive signaling networks function across different cell types, tissues, and developmental contexts [108,109,110]. At the developmental level, quantitative phenotyping combined with high-resolution live imaging will provide powerful tools to explore how temperature and other environmental signals are converted into changes in developmental trajectories [111,112,113].

Beyond enhancing our fundamental understanding of thermosensing and other environmental-signal-mediated developmental plasticity, these advances are likely to open new avenues for rational crop improvement. Meanwhile, progress in precision genome engineering makes it possible to translate mechanistic insights into practical breeding strategies [114]. A recent study showed that precise insertion of heat-responsive cis-elements into the promoter of a key gene involved in carbon-partitioning enabled temperature-dependent transcriptional activation under warm conditions, thereby dynamically adjusting source–sink relationships and improving yield stability in both tomato and rice under elevated temperatures [22]. Importantly, this approach did not constitutively enhance gene expression; rather, it endowed developmental and metabolic pathways with environmentally responsive regulatory plasticity. This suggests that crop improvement under future climates may undergo a conceptual transition from static genetic optimization toward dynamic engineering of developmental programs that actively respond to environmental fluctuations.

Collectively, these advances are deepening our understanding of how plants perceive and respond to moderately elevated temperatures, ranging from the molecular basis of thermosensing to the reprogramming of whole-plant development. A key challenge ahead is to integrate temperature perception, environmental signal integration, and developmental decision-making into a coherent framework that can account for the emergence of plastic responses across diverse tissues, developmental stages, and species. Such knowledge will provide a foundation for designing beneficial developmental plasticity in crops. As global climate change continues to reshape agricultural ecosystems through simultaneous alterations in temperature and other environmental factors, harnessing developmental plasticity may become a critical strategy for safeguarding agricultural productivity and ensuring food security in the future.

## Figures and Tables

**Table 1:** Representative temperature regimes used in studies of different plant species.

Species	Reported Optimal Growth Temperature Range	Moderately Elevated Temperatures Used in Studies	Extreme Heat Temperatures Used in Studies	References
*Arabidopsis*	22 °C	26–28 °C	34–38 °C	[10]
maize	24–28 °C	32–35 °C	42–45 °C	[11,12,13,14]
Rice	25–30 °C	30 °C → 35 °C	37–45 °C	[15,16,17,18]
Tomato	23–25 °C	30 °C → 32 °C	38–45 °C	[19,20,21,22,23]
Barley	15–22 °C	28 °C	33–44 °C	[24,25,26,27,28,29]

Note: Because thermal responses vary substantially among plant species, genotypes, developmental stages, and exposure durations, no universal temperature thresholds define moderately elevated temperatures or extreme heat. Therefore, the temperature ranges listed here represent commonly used experimental regimes in representative studies rather than absolute physiological boundaries.

**Table 2:** Key regulators of developmental plasticity under moderately elevated temperatures.

Regulator	Species	Temperature Regime	Experimental Conditions	Mechanism	Effect on Plasticity	References
Thermal perception						
phyB	*Arabidopsis*; maize; potato	22 °C → 27–28 °C	Growth chamber	Thermo reversion from active Pfr to inactive Pr	Releases the repression of PIFs	[31,32,33,34,35,36,37,44]
ELF3	*Arabidopsis*	22 °C → 27 °C	Growth chamber	Phase separation of prion-like domain	Relieves the transcriptional repression of *PIF4*	[38,39,40,41,42]
*PIF7* mRNA	*Arabidopsis*	17 °C → 27 °C	Growth chamber	Conformational change in 5′ UTR	Enhances the translation efficiency of *PIF7*	[43]
Vegetative growth						
PIF4	*Arabidopsis*; tomato	22 °C → 28 °C	Growth chamber	Promotes the expression of plasticity-related genes	Promotes hypocotyl/petiole elongation, leaf hyponasty, flowering, and reduces stomatal index	[45,46,47,48,49,50]
PIF7	*Arabidopsis*	21 °C → 30 °C	Growth chamber	Activate auxin biosynthesis	Promoting synergistic hypocotyl elongation under moderately elevated temperature and shade conditions	[51]
H2A.Z	*Arabidopsis*	17 °C → 27 °C	Growth chamber	Dissociates from chromatin under moderately elevated temperatures	Inhibits expression of plasticity-related genes	[52]
ARP6	*Arabidopsis*	22 °C → 28 °C	Growth chamber	Promotes H2A.Z deposition at thermoresponsive loci	Inhibits expression of plasticity-related genes	[53]
INO80	*Arabidopsis*	21 °C → 27 °C	Growth chamber	Promotes H2A.Z eviction at thermoresponsive loci; Regulates histone methylation at target genes	Promotes expression of plasticity-related genes	[54,55]
MED25	*Arabidopsis*	22 °C → 27 °C	Growth chamber	Recruits HDA9 to *YUC8* loci	Promotes auxin-dependent thermomorphogenesis	[56]
YUC8, CYP79B2	*Arabidopsis*	22 °C → 29 °C	Growth chamber	Activate auxin biosynthesis	Promote hypocotyl growth	[47]
TAA1	*Arabidopsis*	20 °C → 28 °C	Growth chamber	Activate auxin biosynthesis	Promote hypocotyl growth	[48]
HDA9	*Arabidopsis*	22 °C → 27 °C	Growth chamber	Mediates histone deacetylation and promotes H2A.Z eviction at *YUC8* loci	Promotes auxin-dependent thermomorphogenesis	[57]
MED17	*Arabidopsis*	22 °C → 28 °C	Growth chamber	Promotes H3K4me3 enrichment at thermoresponsive loci	Promotes hypocotyl growth	[58]
JMJ14, JMJ15	*Arabidopsis*	20 °C → 27 °C	Growth chamber	Regulate H3K4me3 at thermoresponsive loci	Promote expression of plasticity-related genes	[59]
PIN3/4/7 D6PK	*Arabidopsis*	20 °C → 28 °C	Growth chamber	Regulate polar auxin flow	Regulate hypocotyl growth	[60]
SEU	*Arabidopsis*	22 °C → 28 °C	Growth chamber	Promotes auxin biosynthetic and responsive genes; Affects H3K4me3 at *IAA6* and *IAA19* genes	Promotes hypocotyl growth	[61]
MRG2	*Arabidopsis*	22 °C → 28 °C	Growth chamber	Stabilizes PIF4 protein and enhance H4K5ac at thermoresponsive genes	Promotes hypocotyl growth	[62]
SlHDA4	Tomato	23 °C → 30 °C	Growth chamber	Removes H3K9ac at auxin biosynthesis and signaling genes to repress their expression	Suppresses lateral bud outgrowth	[19]
FERONIA	*Arabidopsis*	22 °C → 28 °C	Growth chamber	Regulates auxin signaling	Promotes hypocotyl growth	[63]
PIN1/2	*Arabidopsis*	20 °C → 28 °C	Growth chamber	Regulate polar auxin flow	Regulate root growth	[64,65]
PILS6	*Arabidopsis*	21 °C → 29 °C	Growth chamber	Regulate polar auxin flow	Regulate root growth	[66]
BZR1	*Arabidopsis*	20 °C → 28 °C	Growth chamber	Interacts with PIF4 and ARF6 and activates *PIF4* expression	Promotes hypocotyl elongation	[67,68,69]
SIDWF4	Tomato	25 °C → 32 °C	Growth chamber	Activates brassinosteroids biosynthesis	Promotes hypocotyl elongation	[21]
DELLA	*Arabidopsis*	22 °C → 28 °C	Growth chamber	Interacts with PIF4, ARF6, and BZR1 to inhibit their DNA-binding activity	Inhibits hypocotyl elongation	[34,68,70,71]
EIN3	*Arabidopsis*	22 °C → 28 °C	Growth chamber	Induces *APD7* expression to inactivate plasma membrane H^+^-ATPase	Attenuates auxin-induced cell expansion	[72]
JAZ3	*Arabidopsis*	22 °C → 28 °C	Growth chamber	Inhibits PIF4 activity	Inhibits hypocotyl elongation	[73]
Reproductive growth						
HvMADS1, HvCKX3	Barley	15 °C/10 °C → 28 °C/23 °C	Growth chamber	Maintain cytokinin homeostasis	Constrain plasticity of spikelet development	[24]
TAP	Rice	22 °C → 28 °C → 30 °C → 34 °C	Growth chamber	Regulates the expression of panicle and spikelet development genes	Constrains plasticity of inflorescence and spikelet	[17]
MADS8	Barley, Rice	Barley: 15 °C/10 °C → 28 °C/23 °C Rice: 28 °C → 34 °C	Growth chamber	Activates D-class floral homeotic genes	Constrains plasticity of floral organ development	[25]
NDL1	Maize	24 °C/20 °C → 32 °C/28 °C	Field trial and Growth chamber	Maintains mitochondrial redox balance and auxin homeostasis	Constrains plasticity of tassel and spikelet development	[12]
ZmRNRL1	Maize	28 °C/21 °C → 34.9 °C/26 °C	Field trial and Growth chamber	Maintains dNTP pools for DNA synthesis	Constrains plasticity of tassel development	[11]
EG1	Rice	25 °C/20 °C → 35 °C/20 °C	Field trial and Growth chamber	Regulates mitochondrial lipid homeostasis	Constrains plasticity of floral organ development	[15]
PPD-H1	Barley	20 °C/16 °C → 28 °C/24 °C	Growth chamber	Balances hormonal pathways	Constrains plasticity of floral organ development	[26]
cpHSP70-2 /OsHsp40-1	Rice	28 °C/24 °C → 34 °C/28 °C	Field trial and Growth chamber	Stabilizes key enzymes of starch biosynthesis	Constrains plasticity of grain development	[74,75]

## Data Availability

No new data were created or analyzed in this study. Data sharing is not applicable to this article.

## References

[1] Pagnussat G.C., Gomez-Casati D.F. (2024). Plant development and reproduction in a changing environment. J. Exp. Bot..

[2] Zhu J., Cao X., Deng X. (2023). Epigenetic and transcription factors synergistically promote the high temperature response in plants. Trends Biochem. Sci..

[3] Fehér A., Hamid R.S.B., Magyar Z. (2025). How do *Arabidopsis* seedlings sense and react to increasing ambient temperatures?. Plants.

[4] Reis R.S., Manavella P. (2023). Thermomorphogenesis: Opportunities and challenges in post-transcriptional regulation. J. Exp. Bot..

[5] Lee H., Romero J., IPCC (2023). Summary for Policymakers. Climate Change 2023: Synthesis Report. Contribution of Working Groups I, II and III to the Sixth Assessment Report of the Intergovernmental Panel on Climate Change.

[6] Wang F., Zhan C., Zou L. (2023). Risk of crop yield reduction in China under 1.5 °C and 2 °C global warming from CMIP6 models. Foods.

[7] Zhao C., Liu B., Piao S., Wang X., Lobell D.B., Huang Y., Huang M., Yao Y., Bassu S., Ciais P. (2017). Temperature increase reduces global yields of major crops in four independent estimates. Proc. Natl. Acad. Sci. USA.

[8] Kan Y., Mu X.R., Gao J., Lin H.X., Lin Y. (2023). The molecular basis of heat stress responses in plants. Mol. Plant.

[9] Zhu T., Fonseca De Lima C.F., De Smet I., Penfield S. (2021). The heat is on: How crop growth, development, and yield respond to high temperature. J. Exp. Bot..

[10] Wang J., Lai Z., Lei Z., Chang F. (2026). Comprehensive analysis of RNA expression and isoform diversity reveals molecular mechanisms in plant responses to diverse thermal conditions. Phenomics.

[11] Xie S., Luo H., Huang Y., Wang Y., Ru W., Shi Y., Huang W., Wang H., Dong Z., Jin W. (2020). A missense mutation in a large subunit of ribonucleotide reductase confers temperature-gated tassel formation. Plant Physiol..

[12] Liu Q., Galli M., Liu X., Federici S., Buck A., Cody J., Labra M., Gallavotti A. (2019). *NEEDLE1* encodes a mitochondria localized ATP-dependent metalloprotease required for thermotolerant maize growth. Proc. Natl. Acad. Sci. USA.

[13] Li Z., Li Z., Ji Y., Wang C., Wang S., Shi Y., Le J., Zhang M. (2024). The heat shock factor 20-HSF4-cellulose synthase A2 module regulates heat stress tolerance in maize. Plant Cell.

[14] Ma L., Zhang Z., Liu M., Wang Z., Zhang X., Guo X., Zhang J., Hu J., Zhu W., Li Q. (2025). Manipulation of the central autophagy component ZmATG8c affects thermotolerance in maize. Plant Physiol..

[15] Zhang B., Wu S., Zhang Y., Xu T., Guo F., Tang H., Li X., Wang P., Qian W., Xue Y. (2016). A high temperature-dependent mitochondrial lipase EXTRA GLUME1 promotes floral phenotypic robustness against temperature fluctuation in rice (*Oryza sativa* L.). PLoS Genet..

[16] Lu H.P., Liu X.H., Wang M.J., Zhu Q.Y., Lyu Y.S., Xu J.H., Liu J.X. (2025). The NAT1-bHLH110-CER1/CER1L module regulates heat stress tolerance in rice. Nat. Genet..

[17] Zhang P., Zhu W., He Y., Fan J., Shi J., Fu R., Hu J., Li L., Zhang D., Liang W. (2022). THERMO-SENSITIVE BARREN PANICLE (TAP) is required for rice panicle and spikelet development at high ambient temperature. New Phytol..

[18] Kan Y., Mu X.R., Zhang H., Gao J., Shan J.X., Ye W.W., Lin H.X. (2022). TT2 controls rice thermotolerance through SCT1-dependent alteration of wax biosynthesis. Nat. Plants.

[19] Song X., Liu Y., Meng X., Chen S., Sang K., Zheng Q., Zhou Y., Considine M., Yu J., Xia X. (2025). Warm temperature activates the TCP15-HDA4 module to suppress shoot branching through promoting auxin biosynthesis and signaling in tomato. Mol. Plant.

[20] Zhu L., Liu Z., Jin Y., Xu T., Wang K., Qi Z., Sobieszczuk-Nowicka E., Fotopoulos V., Yu J., Zhou J. (2026). The E3 ubiquitin ligases SlXERICO1 and 3 regulate thermotolerance in *Solanum lycopersicum*. Plant Cell.

[21] Zhu C., Hu Z., Hu C., Ma H., Zhou J., Xia X., Shi K., Foyer C.H., Yu J., Zhou Y. (2024). SlCPK27 cross-links SlHY5 and SlPIF4 in brassinosteroid-dependent photo- and thermo-morphogenesis in tomato. Proc. Natl. Acad. Sci. USA.

[22] Lou H., Li S., Shi Z., Zou Y., Zhang Y., Huang X., Yang D., Yang Y., Li Z., Xu C. (2025). Engineering source-sink relations by prime editing confers heat-stress resilience in tomato and rice. Cell.

[23] Huang Z., Lin R., Dong Y., Tang M., Xia X., Fang L., Yu J., Kang H., Zhou Y. (2025). *MiR164a*-targeted NAM3 inhibits thermotolerance in tomato by regulating HSFA4b-mediated redox homeostasis. Plant Physiol..

[24] Li G., Kuijer H.N.J., Yang X., Liu H., Shen C., Shi J., Betts N., Tucker M.R., Liang W., Waugh R. (2021). MADS1 maintains barley spike morphology at high ambient temperatures. Nat. Plants.

[25] Shen C., Zhang Y., Li G., Shi J., Wang D., Zhu W., Yang X., Dreni L., Tucker M.R., Zhang D. (2024). MADS8 is indispensable for female reproductive development at high ambient temperatures in cereal crops. Plant Cell.

[26] Lan T., Walla A., Çolpan Karışan K.E., Buchmann G., Wewer V., Metzger S., Vardanega I., Haraldsson E.B., Helmsorig G., Thirulogachandar V. (2025). PHOTOPERIOD 1 enhances stress resistance and energy metabolism to promote spike fertility in barley under high ambient temperatures. Plant Physiol..

[27] Fernandez E.C., Tu G., Dai W., Yang S., Liu Z., Grzybowski M., Liang Z. (2026). Spatial coordination between leaf gradient and temperature response in barley. Plant J..

[28] Chaudhary R., Baliyan S., Khungar L., Sirohi P., Singh S., Mishra S.K., Rajkumar M.S., Saini S.S., Germain H., Sircar D. (2026). Overexpression of barley heat stress transcription factor HvHsfA6a provides thermotolerance by modulating transcriptome and metabolome. Plant J..

[29] Pratx L., Dakhiya Y., Nissen R., Purushotham P., Hoffie I., Kumlehn J., Kappel C., Bäurle I. (2025). Conserved heat shock factors HvHSFA2 and HvHSFA3 control barley heat stress memory through diverged mechanisms. Nat. Commun..

[30] Ludwig W., Hayes S., Trenner J., Delker C., Quint M. (2021). On the evolution of plant thermomorphogenesis. J. Exp. Bot..

[31] Legris M., Nieto C., Sellaro R., Prat S., Casal J.J. (2017). Perception and signalling of light and temperature cues in plants. Plant J..

[32] Rockwell N.C., Su Y.S., Lagarias J.C. (2006). Phytochrome structure and signaling mechanisms. Annu. Rev. Plant Biol..

[33] Kerbler S.M., Wigge P.A. (2023). Temperature sensing in plants. Annu. Rev. Plant Biol..

[34] de Lucas M., Davière J.M., Rodríguez-Falcón M., Pontin M., Iglesias-Pedraz J.M., Lorrain S., Fankhauser C., Blázquez M.A., Titarenko E., Prat S. (2008). A molecular framework for light and gibberellin control of cell elongation. Nature.

[35] Lorrain S., Allen T., Duek P.D., Whitelam G.C., Fankhauser C. (2007). Phytochrome-mediated inhibition of shade avoidance involves degradation of growth-promoting bHLH transcription factors. Plant J..

[36] Jung J.H., Domijan M., Klose C., Biswas S., Ezer D., Gao M., Khattak A.K., Box M.S., Charoensawan V., Cortijo S. (2016). Phytochromes function as thermosensors in *Arabidopsis*. Science.

[37] Legris M., Klose C., Burgie E.S., Rojas C.C.R., Neme M., Hiltbrunner A., Wigge P.A., Schäfer E., Vierstra R.D., Casal J.J. (2016). Phytochrome B integrates light and temperature signals in *Arabidopsis*. Science.

[38] Ezer D., Jung J.H., Lan H., Biswas S., Gregoire L., Box M.S., Charoensawan V., Cortijo S., Lai X., Stockle D. (2017). The evening complex coordinates environmental and endogenous signals in *Arabidopsis*. Nat. Plants.

[39] Nusinow D.A., Helfer A., Hamilton E.E., King J.J., Imaizumi T., Schultz T.F., Farre E.M., Kay S.A. (2011). The ELF4-ELF3-LUX complex links the circadian clock to diurnal control of hypocotyl growth. Nature.

[40] Jung J.H., Barbosa A.D., Hutin S., Kumita J.R., Gao M., Derwort D., Silva C.S., Lai X., Pierre E., Geng F. (2020). A prion-like domain in ELF3 functions as a thermosensor in *Arabidopsis*. Nature.

[41] Hutin S., Kumita J.R., Strotmann V.I., Dolata A., Ling W.L., Louafi N., Popov A., Milhiet P.-E., Blackledge M., Nanao M.H. (2023). Phase separation and molecular ordering of the prion-like domain of the *Arabidopsis* thermosensory protein EARLY FLOWERING 3. Proc. Natl. Acad. Sci. USA.

[42] Silva C.S., Nayak A., Lai X., Hutin S., Hugouvieux V., Jung J.-H., López-Vidriero I., Franco-Zorrilla J.M., Panigrahi K.C.S., Nanao M.H. (2020). Molecular mechanisms of Evening Complex activity in *Arabidopsis*. Proc. Natl. Acad. Sci. USA.

[43] Chung B.Y.W., Balcerowicz M., Di Antonio M., Jaeger K.E., Geng F., Franaszek K., Marriott P., Brierley I., Firth A.E., Wigge P.A. (2020). An RNA thermoswitch regulates daytime growth in *Arabidopsis*. Nat. Plants.

[44] Burgie E.S., Gannam Z.T.K., McLoughlin K.E., Sherman C.D., Holehouse A.S., Stankey R.J., Vierstra R.D. (2021). Differing biophysical properties underpin the unique signaling potentials within the plant phytochrome photoreceptor families. Proc. Natl. Acad. Sci. USA.

[45] Kumar S.V., Lucyshyn D., Jaeger K.E., Alós E., Alvey E., Harberd N.P., Wigge P.A. (2012). Transcription factor PIF4 controls the thermosensory activation of flowering. Nature.

[46] Lau O.S., Song Z., Zhou Z., Davies K.A., Chang J., Yang X., Wang S., Lucyshyn D., Tay I.H.Z., Wigge P.A. (2018). Direct control of *SPEECHLESS* by PIF4 in the high-temperature response of stomatal development. Curr. Biol..

[47] Sun J., Qi L., Li Y., Chu J., Li C. (2012). PIF4-mediated activation of YUCCA8 expression integrates temperature into the Auxin pathway in regulating *Arabidopsis* hypocotyl growth. PLoS Genet..

[48] Franklin K.A., Lee S.H., Patel D., Kumar S.V., Spartz A.K., Gu C., Ye S., Yu P., Breen G., Cohen J.D. (2011). PHYTOCHROME-INTERACTING FACTOR 4 (PIF4) regulates auxin biosynthesis at high temperature. Proc. Natl. Acad. Sci. USA.

[49] Zhou D., Wang X., Wang X., Mao T. (2023). PHYTOCHROME INTERACTING FACTOR 4 regulates microtubule organization to mediate high temperature-induced hypocotyl elongation in *Arabidopsis*. Plant Cell.

[50] Park Y.J., Lee H.J., Gil K.E., Kim J.Y., Lee J.H., Lee H., Cho H.T., Vu L.D., De Smet I., Park C.M. (2019). Developmental programming of thermonastic leaf movement. Plant Physiol..

[51] Burko Y., Willige B.C., Seluzicki A., Novak O., Ljung K., Chory J. (2022). PIF7 is a master regulator of thermomorphogenesis in shade. Nat. Commun..

[52] Kumar S.V., Wigge P.A. (2010). H2A.Z-containing nucleosomes mediate the thermosensory response in *Arabidopsis*. Cell.

[53] Verma N., Singh D., Mittal L., Banerjee G., Noryang S., Sinha A.K. (2024). MPK4-mediated phosphorylation of PHYTOCHROME INTERACTING FACTOR4 controls thermosensing by regulating histone variant H2A.Z deposition. Plant Cell.

[54] Xue M., Zhang H., Zhao F., Zhao T., Li H., Jiang D. (2021). The INO80 chromatin remodeling complex promotes thermomorphogenesis by connecting H2A.Z eviction and active transcription in *Arabidopsis*. Mol. Plant.

[55] Shao Z., Bai Y., Huq E., Qiao H. (2024). LHP1 and INO80 cooperate with ethylene signaling for warm ambient temperature response by activating specific bivalent genes. Cell Rep..

[56] Shapulatov U., van Zanten M., van Hoogdalem M., Meisenburg M., van Hall A., Kappers I., Fasano C., Facella P., Loh C.C., Perrella G. (2023). The Mediator complex subunit MED25 interacts with HDA9 and PIF4 to regulate thermomorphogenesis. Plant Physiol..

[57] van der Woude L.C., Perrella G., Snoek B.L., van Hoogdalem M., Novák O., van Verk M.C., van Kooten H.N., Zorn L.E., Tonckens R., Dongus J.A. (2019). HISTONE DEACETYLASE 9 stimulates auxin-dependent thermomorphogenesis in *Arabidopsis thaliana* by mediating H2A.Z depletion. Proc. Natl. Acad. Sci. USA.

[58] Agrawal R., Sharma M., Dwivedi N., Maji S., Thakur P., Junaid A., Fajkus J., Laxmi A., Thakur J.K. (2022). MEDIATOR SUBUNIT17 integrates jasmonate and auxin signaling pathways to regulate thermomorphogenesis. Plant Physiol..

[59] Cui X., Zheng Y., Lu Y., Issakidis-Bourguet E., Zhou D.X. (2021). Metabolic control of histone demethylase activity involved in plant response to high temperature. Plant Physiol..

[60] Bellstaedt J., Trenner J., Lippmann R., Poeschl Y., Zhang X., Friml J., Quint M., Delker C. (2019). A mobile auxin signal connects temperature sensing in cotyledons with growth responses in hypocotyls. Plant Physiol..

[61] Huai J., Zhang X., Li J., Ma T., Zha P., Jing Y., Lin R. (2018). SEUSS and PIF4 coordinately regulate light and temperature signaling pathways to control plant growth. Mol. Plant.

[62] Zhou N., Li C., Xie W., Liang N., Wang J., Wang B., Wu J., Shen W.H., Liu B., Dong A. (2024). Histone methylation readers MRG1/2 interact with PIF4 to promote thermomorphogenesis in *Arabidopsis*. Cell Rep..

[63] Wang K., Yan H., Guo X., Lin Q., Li J., Peng A., Fu Y., Gong Z., Yang S., Ding Y. (2026). FERONIA orchestrates plasma membrane nanoclusters for plant thermotolerance. Science.

[64] Ai H., Bellstaedt J., Bartusch K.S., Eschen-Lippold L., Babben S., Balcke G.U., Tissier A., Hause B., Andersen T.G., Delker C. (2023). Auxin-dependent regulation of cell division rates governs root thermomorphogenesis. EMBO J..

[65] Hanzawa T., Shibasaki K., Numata T., Kawamura Y., Gaude T., Rahman A. (2013). Cellular auxin homeostasis under high temperature is regulated through a sorting NEXIN1-dependent endosomal trafficking pathway. Plant Cell.

[66] Feraru E., Feraru M.I., Barbez E., Waidmann S., Sun L., Gaidora A., Kleine-Vehn J. (2019). PILS6 is a temperature-sensitive regulator of nuclear auxin input and organ growth in *Arabidopsis thaliana*. Proc. Natl. Acad. Sci. USA.

[67] Oh E., Zhu J.Y., Wang Z.Y. (2012). Interaction between BZR1 and PIF4 integrates brassinosteroid and environmental responses. Nat. Cell Biol..

[68] Oh E., Zhu J.Y., Bai M.Y., Arenhart R.A., Sun Y., Wang Z.Y. (2014). Cell elongation is regulated through a central circuit of interacting transcription factors in the *Arabidopsis* hypocotyl. eLife.

[69] Ibañez C., Delker C., Martinez C., Bürstenbinder K., Janitza P., Lippmann R., Ludwig W., Sun H., James G.V., Klecker M. (2018). Brassinosteroids dominate hormonal regulation of plant thermomorphogenesis via BZR1. Curr. Biol..

[70] Gallego-Bartolomé J., Minguet E.G., Grau-Enguix F., Abbas M., Locascio A., Thomas S.G., Alabadí D., Blázquez M.A. (2012). Molecular mechanism for the interaction between gibberellin and brassinosteroid signaling pathways in *Arabidopsis*. Proc. Natl. Acad. Sci. USA.

[71] Li Q.F., Wang C., Jiang L., Li S., Sun S.S.M., He J.X. (2012). An interaction between BZR1 and DELLAs mediates direct signaling crosstalk between brassinosteroids and gibberellins in *Arabidopsis*. Sci. Signal..

[72] Kim J.Y., Park Y.J., Lee J.H., Kim Z.H., Park C.M. (2021). EIN3-mediated ethylene signaling attenuates auxin response during hypocotyl thermomorphogenesis. Plant Cell Physiol..

[73] Huai J., Gao N., Yao Y., Du Y., Guo Q., Lin R. (2024). JASMONATE ZIM-domain protein 3 regulates photomorphogenesis and thermomorphogenesis through inhibiting PIF4 in *Arabidopsis*. Plant Physiol..

[74] Tabassum R., Dosaka T., Ichida H., Morita R., Ding Y., Abe T., Katsube-Tanaka T. (2020). *FLOURY ENDOSPERM11-2* encodes plastid HSP70-2 involved with the temperature-dependent chalkiness of rice (*Oryza sativa* L.) grains. Plant J..

[75] Lu F., Jiao G., Qiu J., Zhao S., Zhao F., Wang P., Chen L., Chen P., Li X., Dong N. (2025). A molecular module improves rice grain quality and yield at high temperatures. Natl. Sci. Rev..

[76] Alseekh S., Klemmer A., Yan J., Guo T., Fernie A.R. (2025). Embracing plant plasticity or robustness as a means of ensuring food security. Nat. Commun..

[77] Seth P., Sebastian J. (2024). Plants and global warming: Challenges and strategies for a warming world. Plant Cell Rep..

[78] Xiong S.X., Zhong X., Jia Q.S., Yang N.Y., Han Y., Zhang Z.L., Xu X.F., Zhu J., Zhang C., Yang Z.N. (2025). TEK and other AHL proteins interact with TCP14/15 to regulate pollen wall formation in *Arabidopsis*. Plant J..

[79] Gray W.M., Östin A., Sandberg G., Romano C.P., Estelle M. (1998). High temperature promotes auxin-mediated hypocotyl elongation in *Arabidopsis*. Proc. Natl. Acad. Sci. USA.

[80] Koini M.A., Alvey L., Allen T., Tilley C.A., Harberd N.P., Whitelam G.C., Franklin K.A. (2009). High temperature-mediated adaptations in plant architecture require the bHLH transcription factor PIF4. Curr. Biol..

[81] Crawford A.J., McLachlan D.H., Hetherington A.M., Franklin K.A. (2012). High temperature exposure increases plant cooling capacity. Curr. Biol..

[82] Ren C., Bodendorf J., Moreno-Risueno M.A., Kleine-Vehn J. (2024). Two distinct oscillatory auxin signals define the plasticity of lateral rooting in *Arabidopsis thaliana*. bioRxiv.

[83] Fonseca de Lima C.F., Kleine-Vehn J., De Smet I., Feraru E. (2021). Getting to the root of belowground high temperature responses in plants. J. Exp. Bot..

[84] Bawa G., Feng L., Chen G., Chen H., Hu Y., Pu T., Cheng Y., Shi J., Xiao T., Zhou W. (2020). Gibberellins and auxin regulate soybean hypocotyl elongation under low light and high-temperature interaction. Physiol. Plant..

[85] Vu L.D., Xu X., Zhu T., Pan L., van Zanten M., de Jong D., Wang Y., Vanremoortele T., Locke A.M., van de Cotte B. (2021). The membrane-localized protein kinase MAP4K4/TOT3 regulates thermomorphogenesis. Nat. Commun..

[86] Sang Q., Fan L., Liu T., Qiu Y., Du J., Mo B., Chen M., Chen X. (2023). MicroRNA156 conditions auxin sensitivity to enable growth plasticity in response to environmental changes in *Arabidopsis*. Nat. Commun..

[87] March-Díaz R., Reyes J.C. (2009). The beauty of being a variant: H2A.Z and the SWR1 complex in plants. Mol. Plant.

[88] Kim J., Bordiya Y., Xi Y., Zhao B., Kim D.-H., Pyo Y., Zong W., Ricci W.A., Sung S. (2023). Warm temperature-triggered developmental reprogramming requires VIL1-mediated, genome-wide H3K27me3 accumulation in *Arabidopsis*. Development.

[89] Martínez C., Espinosa-Ruíz A., de Lucas M., Bernardo-García S., Franco-Zorrilla J.M., Prat S. (2018). PIF4-induced BR synthesis is critical to diurnal and thermomorphogenic growth. EMBO J..

[90] Zhu Q.Y., Wang M.J., Luo H.D., Zhang L.L., Lu H.P., Liu J.X. (2025). The HECT-family protein UPL3 suppresses thermomorphogenesis by modulating BZR1 accumulation. Plant J..

[91] Stavang J.A., Gallego-Bartolome J., Gomez M.D., Yoshida S., Asami T., Olsen J.E., Garcia-Martinez J.L., Alabadi D., Blazquez M.A. (2009). Hormonal regulation of temperature-induced growth in *Arabidopsis*. Plant J..

[92] Bai M.Y., Shang J.X., Oh E., Fan M., Bai Y., Zentella R., Sun T.P., Wang Z.Y. (2012). Brassinosteroid, gibberellin and phytochrome impinge on a common transcription module in *Arabidopsis*. Nat. Cell Biol..

[93] Casal J.J., Balasubramanian S. (2019). Thermomorphogenesis. Annu. Rev. Plant Biol..

[94] Quint M., Delker C., Franklin K.A., Wigge P.A., Halliday K.J., van Zanten M. (2016). Molecular and genetic control of plant thermomorphogenesis. Nat. Plants.

[95] Delker C., Quint M., Wigge P.A. (2022). Recent advances in understanding thermomorphogenesis signaling. Curr. Opin. Plant Biol..

[96] Jacob T., Maciel Rodrigues Junior O., Quint M. (2025). Hormonal regulation of root growth under moderately elevated temperatures. Ann. Bot..

[97] Shi W., Muthurajan R., Rahman H., Selvam J., Peng S., Zou Y., Jagadish K.S.V. (2013). Source-sink dynamics and proteomic reprogramming under elevated night temperature and their impact on rice yield and grain quality. New Phytol..

[98] Sun S., Liu Z., Wang X., Song J., Fang S., Kong J., Li R., Wang H., Cui X. (2024). Genetic control of thermomorphogenesis in tomato inflorescences. Nat. Commun..

[99] Zhang N., Liu Y., Gui S., Wang Y. (2025). Regulation of tillering and panicle branching in rice and wheat. J. Genet. Genom..

[100] Takai T., Byrne M. (2024). Potential of rice tillering for sustainable food production. J. Exp. Bot..

[101] Adriani D.E., Lafarge T., Dardou A., Fabro A., Clément-Vidal A., Yahya S., Dingkuhn M., Luquet D. (2016). The qTSN positive effect on panicle and flag leaf size of rice is associated with an early down-regulation of tillering. Front. Plant Sci..

[102] Yoshida S. (1973). Effects of temperature on growth of the rice plant (*Oryza sativa* L.) in a controlled environment. Soil. Sci. Plant Nutr..

[103] Song R., Xue M., Zhang H., Li X., Li H., Jiang D. (2025). Drought inhibits thermomorphogenesis via salicylic acid-mediated suppression of ELF3 phase separation. Plant J..

[104] Lee S., Showalter J., Zhang L., Cassin-Ross G., Rouached H., Busch W. (2024). Nutrient levels control root growth responses to high ambient temperature in plants. Nat. Commun..

[105] Hwang G., Lee T., Park J., Paik I., Lee N., Kim Y.J., Song Y.H., Kim W.Y., Oh E. (2025). UV-B increases active phytochrome B to suppress thermomorphogenesis and enhance UV-B stress tolerance at high temperatures. Plant Commun..

[106] Ma D., Li X., Guo Y., Chu J., Fang S., Yan C., Noel J.P., Liu H. (2016). Cryptochrome 1 interacts with PIF4 to regulate high temperature-mediated hypocotyl elongation in response to blue light. Proc. Natl. Acad. Sci. USA.

[107] Abramson J., Adler J., Dunger J., Evans R., Green T., Pritzel A., Ronneberger O., Willmore L., Ballard A.J., Bambrick J. (2024). Accurate structure prediction of biomolecular interactions with AlphaFold 3. Nature.

[108] Bennett H.M., Stephenson W., Rose C.M., Darmanis S. (2023). Single-cell proteomics enabled by next-generation sequencing or mass spectrometry. Nat. Methods.

[109] Kang M., Vu A.H., Casper A.L., Kim R., Wurlitzer J., Heinicke S., Yeroslaviz A., Caputi L., O’Connor S.E. (2025). Single-cell metabolome and RNA-seq multiplexing on single plant cells. Proc. Natl. Acad. Sci. USA.

[110] Lee T.A., Illouz-Eliaz N., Nobori T., Xu J., Jow B., Nery J.R., Ecker J.R. (2025). A single-cell, spatial transcriptomic atlas of the *Arabidopsis* life cycle. Nat. Plants.

[111] Fahlgren N., Feldman M., Gehan M.A., Wilson M.S., Shyu C., Bryant D.W., Hill S.T., McEntee C.J., Warnasooriya S.N., Kumar I. (2015). A versatile phenotyping system and analytics platform reveals diverse temporal responses to water availability in *Setaria*. Mol. Plant.

[112] Ovecka M., von Wangenheim D., Tomancak P., Samajova O., Komis G., Samaj J. (2018). Multiscale imaging of plant development by light-sheet fluorescence microscopy. Nat. Plants.

[113] Yang W., Duan L., Chen G., Xiong L., Liu Q. (2013). Plant phenomics and high-throughput phenotyping: Accelerating rice functional genomics using multidisciplinary technologies. Curr. Opin. Plant Biol..

[114] Li B., Sun C., Li J., Gao C. (2024). Targeted genome-modification tools and their advanced applications in crop breeding. Nat. Rev. Genet..

